# Epidemiology of arthritis, chronic back pain, gout, osteoporosis, spondyloarthropathies and rheumatoid arthritis among 1.5 million patients in Australian general practice: NPS MedicineWise MedicineInsight dataset

**DOI:** 10.1186/s12891-018-1941-x

**Published:** 2018-01-18

**Authors:** David Alejandro González-Chica, Simon Vanlint, Elizabeth Hoon, Nigel Stocks

**Affiliations:** 1 Discipline of General Practice, Adelaide Medical School, NHMRC Centre of Research Excellence to Reduce Inequality in Heart Disease, The University of Adelaide, Adelaide, SA Australia; 2 School of Public Health, The University of Adelaide, Adelaide, SA Australia

**Keywords:** Pain, chronic, Back pain, Musculoskeletal, Arthritis, Epidemiology, Population health

## Abstract

**Background:**

Previous estimates for the prevalence of musculoskeletal conditions (MSK) and chronic pain in Australia have been based on self-report. We aimed to determine the prevalence and distribution of arthritis, chronic back pain, gout, osteoporosis, spondyloarthropathies and rheumatoid arthritis and current consultations for chronic pain among adults attending Australian general practice, and describe their distribution according to sociodemographic characteristics and presence of co-morbidities.

**Methods:**

We investigated 1,501,267 active adult patients (57.6% females; 22.5% ≥65y) evaluated between 2013 and 2016 and included in the MedicineInsight database (a National Prescribing Service MedicineWise program), a large general practice data program that extracts longitudinal de-identified electronic medical record data from ‘active’ patients in over 550 practices. Three main groups of outcomes were investigated: 1) “prevalence” of arthritis, chronic back pain, gout, osteoporosis, spondyloarthropathies, and/or rheumatoid arthritis between 2000 and 2016; 2) “current” diagnosis/encounter for the same conditions occurring between 2013 and 2016, and; 3) “current” consultations for chronic pain of any type occurring between 2013 and 2016.

**Results:**

The combined “prevalence” of the investigated MSK (diagnosis between 2000 and 2016) among adults attending Australian general practice was 16.8% (95%CI 15.9;17.7) with 21.3% (95%CI 20.2;22.4) of the sample consulting for chronic pain between 2013 and 2016. The investigated MSK with the highest “prevalence” were arthritis (9.5%) and chronic back pain (6.7%). Patients with some of these MSK attended general practices more frequently than those without these conditions (median 2.0 and 1.0 contacts/year, respectively). The “prevalence” of the investigated MSK and “current” consultations for chronic pain increased with age, especially in women, but chronic pain remained stable at 22% for males aged > 40 years. The investigated MSK and chronic pain were more frequent among those in lower socioeconomic groups, veterans, Aboriginal and Torrent Strait Islanders, current and ex-smokers, and patients with chronic obstructive pulmonary disease or heart failure.

**Conclusions:**

The investigated MSK are more frequent among lower socioeconomic groups and the elderly. Based on information collected from adults attending Australian general practices, MedicineInsight provided similar estimates to those obtained from population-based studies, with the advantage of being based on medical diagnosis and including a national sample.

**Electronic supplementary material:**

The online version of this article (10.1186/s12891-018-1941-x) contains supplementary material, which is available to authorized users.

## Background

Musculoskeletal conditions (MSK) and chronic pain represent an increasing public health problem worldwide [1–5]. According to the Australian Institute of Health and Welfare (AIHW) and the National Health Survey (NHS), in 2011 they affected 28% of Australian adults (6.1 million) [6]. Due to their chronicity and impact on health status and quality of life, they represent the fourth most expensive group of diseases in Australia, accounting for 9% of total health-care expenditure related to hospitalisations, out-of-hospital health care, and prescribed medications (approximately AUD$5.7 billion of the AUD$65 billion spent for all diseases) [7]. In terms of health service use, in 2010 they were among the ten most frequent problems managed by general practitioners (GPs) (2.7 per 100 encounters for osteoarthritis and 2.6 for back pain), only after metabolic conditions and depression [8].

A few surveys have used self-reported data to investigate the prevalence of these conditions among Australian adults [6, 9, 10], while the Bettering the Evaluation and Care of Health (BEACH) program reported encounter rates and medication use for MSK and chronic pain in adults attending a random sample of Australian general practices [11, 12]. Estimates for MSK and chronic pain vary across studies and information bias has been highlighted as one of the reasons for these differences [13–15]. Self-report for chronic pain and some MSK is less accurate than for sociodemographic characteristics, lifestyle, or other chronic conditions, thus affecting the ability of population-based surveys to provide accurate estimates for the total burden of MSK [14, 15]. On the other hand, studies on this topic using nationally representative samples and based on medical diagnosis are scarce in the scientific literature [3–5, 13].

The Australian Government have recognised MSK and chronic pain as a major public health priority, and emphasised the importance of national real-time data for monitoring their prevalence, management, and adverse effects of medications [16]. More generally, increasing the scope and number of quality improvement activties has been recognised as being important for the Australian health care system and so the National Prescribing Service (NPS) MedicineWise was funded in 2011 to periodically collect longitudinal clinical and prescribing data from Australian general practices through MedicineInsight [17]. Utilising this large ongoing dataset, we aimed to investigate the prevalence of some MSK conditions (arthritis, chronic back pain, gout, osteoporosis, spondyloarthropathies, and rheumatoid arthritis) and consultations for chronic pain among adults attending Australian general practices, describe their distribution according to patients’ sociodemographic characteristics and examine associations with other chronic non-communicable diseases (NCDs).

## Methods

NPS MedicineWise was established in 1998 as an independent, evidence-based, not-for-profit organisation. It aims to promote the quality use of medicines. In 2011, NPS MedicineWise established MedicineInsight to drive quality improvement activities in general practice and improve primary health care and post marketing surveillance of medicine use in Australia [17]. MedicineInsight uses a data extraction tool (GRHANITE™) developed by the University of Melbourne and NPS MedicineWise, which weekly collects de-identified data from patients electronic medical records and securely transfers the information to NPS MedicineWise [17, 18]. Based on a unique identifying number attributed to everyone, patients within practices are tracked over time, allowing the development of an ongoing longitudinal database.

Patients’ information collected by MedicineInsight include: demographics (gender, ethnicity, indigenous status, year of birth, postcode, suburb), diagnoses, reasons for consultations, medicines prescribed and reasons for prescription, known allergies or drug reactions, pathology test orders and results, imaging test orders, instances of referrals to other healthcare professionals (excluding referral documents), instances of patient assessment and management activities, clinical measurements (temperature, blood pressure, weight, height, waist circumference), and smoking status. For privacy reasons, a complete patient’s listed past medical history (PMH considering the whole patient lifespan) is not collected by MedicineInsight [17]. However, by aggregating the information collected over several years at each general practice (46% of individuals with available electronic medical records prior to the launch of MedicineInsight in 2011), the data extraction tool generates a “partial” clinical history for all active patients based on previous diagnoses, medications, laboratory results, and hospital admissions [17, 19–21].

Depending on the clinical information system available at each general practice, GPs used medical coding vocabularies (i.e. ‘DOCLE’, ‘PYEFINCH’ or ‘ICPC’) to register medical diagnosis, reasons for encounter, and reason for prescription into their systems. Although GPs are required to complete all these fields every time they see a patient, the use of the codes is not mandatory and clinicians can enter medical terms as free text [17].

By the end on 2016, MedicineInsight had recruited 557 Australian general practices. Although a non-random sampling process is used, the sample includes practices from all states/territories (New South Wales (NSW) = 29.5%; Victoria (VIC) = 22.7%; Queensland (QLD) = 20.1%; Western Australia (WA) = 12.2%; Tasmania (TAS) = 7.9%; South Australia (SA) = 3.6%; Australia Capital Territory (ACT) = 2.0%; North Territory (NT) = 2.0%), regions (major cities = 59.0%; inner regional = 24.5%; outer regional = 12.6%; remote/very remote =3.8%), and size (1 GP = 6.9%; 2 GPs = 11.6%; 3–5 GPs = 38.0%; 6–8 GPs = 24.0%; > 8 Gps = 19.5%) [17]. Following recommendations for improving data quality [20–23], only practices established for ≥2 years before the end of the analysis period (no interruptions > 2 months in practice), with recorded data (history item, reason for encounter, or reason for prescription) in ≥10% of encounters, and an average of ≥30 prescriptions/week were included in the analyses. Therefore, we used data collected by MedicineInsight from all active patients (three or more visits to the practice in the past 2 years) attending 4668 GPs (14.0% of the 33,275 GPs registered in Australia in 2014–15) in 329 general practices across Australia (4.7% of all 7035 practices in 2011) [24, 25]. All patients aged 18 and above, with information registered in a MedicineInsight practice between 10/2013 and 06/2016, and considered an active patient were eligible for inclusion [17]. The final dataset included a total of 1,544,303 individuals, who had encounters with general practice (including clinical and administrative face-to-face contacts and phone calls), and 7,411,945 different registered medical diagnoses during that period.

The available information from that three-year period was used to determine the percentage of individuals with (what we call) a “current” consultation for MSK, the percentage with a “current” consultation for chronic pain, and the average number of health contacts per year. However, to obtain more accurate data regarding (what we call) the “prevalence” of the investigated MSK (i.e. diagnosis of arthritis, chronic back pain, gout, osteoporosis, spondyloarthropathies, and/or rheumatoid arthritis) [20–23], all data available in MedicineInsight (including current data and past electronic health records going back to 2000 for most enrolled practices) was used to identify individuals with a history (i.e. “prevalence”) of MSK.

Therefore, based in all available data, two different groups of binary variables were created for the investigated MSK: 1) general “prevalence”, considering the diagnosis at any point since 2000 (i.e. past and current health records), and; 2) “current” diagnosis/encounter for the same disease occurring between 2013 and 2016 (i.e. current health records). Patients with some of the investigated MSK were identified from MedicineInsight databases (for both “prevalence” and “current” diagnosis/encounter) using an algorithm that included all diagnosis or reason for encounter (and their synonyms) related to arthritis (“osteoarthritis”, “arthritis” “polyarthritis”, “arthropathy”, “polyarthralgia”, “arthralgia”, “osteoarthrosis”, “arthrosis”), spondyloarthropathies (“spondyloarthritis”, “spondyloarthropathy”, “spondylolisthesis”, “spondylolysis”), rheumatoid arthritis (including polymyalgia rheumatica), osteoporosis, gout, and/or “chronic back pain”. Whenever a field specified the condition as unconfirmed (i.e. “suspected”, “under investigation”, “???”), acute (i.e. “suppurative”, “bacterial”, “infectious”, etc.), or was just as a “family history” (or only a relative affected by these conditions), patients were considered as negative for that condition.

For “current” chronic pain consultations, only one binary variable (no/yes) was generated for data analysis, considering all consultations/diagnosis that occurred between 2013 and 2016. In that case, the terms used included “chronic pain”, “pain treatment” and synonyms of these terms (e.g. “chronic -algia” and “chronic -ache”). Therefore, considering these terms, “chronic back pain” was included under “chronic pain”, but also as a MSK, as this is consistent with clinical practice in Australia and with reports from the Australian Institute of Health and Welfare [6, 7, 26]. Patients were considered negatives for chronic pain whenever the condition was specified as recent/acute.

The total number of contacts with the general practice between 2013 and 2016 (independent of whether it was or not related to some of the investigated MSK or chronic pain) was also extracted from the database, as well as the date of the first and last consultation (past and “current” health records).

“Current” patient related information was obtained from the same database, including sex (male/ female), age (obtained as groups of 5-year categories), state of residence, rurality (major cities, inner regional, outer regional, or remote Australia), Australian Socio-Economic Indexes for Areas (SEIFA) Index of Relative Socio-economic Advantage and Disadvantage (IRSAD) deciles, pension (none, pensioner or health care card owner, Department of Veterans Affair (DVA)), ethnicity (Aboriginal and Torres Strait Islander yes/no), and smoking status (non-smoker, ex-smoker, current smoker). SEIFA-IRSAD is an indicator of relative economic and social advantage/disadvantage of people and households within an area [27]. The deciles were regrouped into quintiles (higher quintile indicating the respondent resides in a more advantaged area) for analysis.

Finally, the prevalence of some common NCDs and their risk factors were obtained following the same procedures used to identify the “prevalence” of the investigated MSK, considering medical registers since 2000 when available (past and current health records). These conditions included cardiometabolic risk factors (hypertension, diabetes mellitus, chronic kidney disease), cardiovascular diseases (ischaemic heart disease, stroke, heart failure), respiratory diseases (asthma, chronic obstructive pulmonary disease (COPD)), and mental health conditions (depression, anxiety).

### Statistical analysis

Categorical variables were expressed as proportions (%), while median with interquartile range (p25-p75) were used to calculate the average number of health contacts, because of this variable’s asymmetry. Confidence intervals of 95% (95%CI) were also estimated. The association between sociodemographic conditions and smoking status with the “prevalence” of the investigated MSK and chronic pain was expressed as prevalence and prevalence ratios (PR and their respective 95%CI estimated using Poisson regression). All associations with the investigated MSK diseases and “current” consultations for chronic pain were adjusted for sex and age, and *p*-values of heterogeneity were used to indicate those associations where the prevalence of the outcome was different across categories of the investigated sociodemographic variables and smoking status.

All analyses were performed in STATA 14.0 (StataCorp, Texas, USA), considering the clusters (general practices) and weighted to the inverse of the individual’s probability of being in the sample (inverse of the average annual number of contacts with the general practice = 1/average number of contacts per year) [11, 12, 28].

The Human Research Ethics Committee of the University of Adelaide exempted this study of an ethical review, as it used existing and non-identifiable data.

## Results

Of the total 1,544,303 active patients available in the MedicineInsight database, 2.8% (*n* = 43,046) were excluded because there was no recorded diagnosis or reason for encounter for the specified period (10/2013 to 06/2016). The percentage of individuals with missing data was up to 4% across categories of the investigated sociodemographic variables, although a higher frequency was observed in the NT (10.7%).

The final sample included for analysis consisted of 1,501,267 adults (57.6% females; 28.3% aged <35y and 22.5% ≥65y), and 65.2% of them had available records in the MedicineInsight database prior to 2013.

The combined “prevalence” of the investigated MSK (positive for arthritis, chronic back pain, gout, osteoporosis, spondyloarthropathies, and/or rheumatoid arthritis, considering past and “current” health records) in the whole sample was 16.8% (95%CI 15.9;17.7) with 21.3% (95%CI 20.2;22.4) of all patients consulting for chronic pain in the period between 2013 and 2016. Considering both variables together, 9.4% of the sample had some of the investigated MSK and also consulted for chronic pain (Additional file 1: Figure S1). For instance, chronic back pain could be both a MSK and a consultation for chronic pain.

Arthritis was the most “prevalent” of the investigated MSK (9.5%), followed by chronic back pain (6.7%), with 3.7% of the whole sample being affected by two or more MSK (Table 1). Among those with any of the investigated MSK, 67.0% consulted for the same reason in the period between 2013 and 2016, and this frequency was greater (86.1%) among those affected by three or more MSK. “Current” consultations for the same MSK were more frequent among those affected by chronic back pain (89.3%) or rheumatoid arthritis (57.7%). Table 1 also shows that 56.1% of the patients with any of the investigated MSK consulted for chronic pain in the period between 2013 and 2016, and this frequency was 46% higher among those affected by three or more MSK. A “current” consultation for chronic pain was also more likely among those affected by chronic back pain (96.7%), and less frequent among patients with gout (30.9%). The average number of health contacts per year was twice as high among those with any of the investigated MSK compared to those without these conditions, and this number increased progressively with the number of MSK’s in the same individual.

**Table 1:** Prevalence of the investigated musculoskeletal diseases and characteristic related to the management of these conditions. Adults aged 18+ years (*N* = 1,501,267)

	Prevalence (2000–2016)^a^ % (95%CI)	Current consultation for the same problem (2013–2016)^b^ %	Current consultation for chronic pain (2013–2016)^b^ %	Average number of health contacts per year (2013–2016)^b,c^ Median [p25-p75]	Average time in the dataset (years) (2000–2016) ^a^ Median [p25-p75]
Positive for any musculoskeletal disease^d^					
No	83.2 (82.3;84.1)	0.0	14.3	1.0 [1.0–2.0]	3.3 [0.7–8.6]
Yes	16.8 (15.9;17.7)	66.7	56.1	2.0 [1.0–5.5]	6.0 [1.8–12.6]
Number of musculoskeletal diseases^d^					
1	13.1 (12.5;13.7)	67.0	54.9	2.0 [1.0–4.5]	5.5 [1.6–12.1]
2	3.1 (2.8;3.4)	62.5	54.8	3.0 [1.0–8.0]	7.3 [2.5–13.9]
3+	0.6 (0.5;0.7)	86.1	80.0	6.5 [2.0–12.5]	8.1 [3.0–14.7]
Type of musculoskeletal disease					
Arthritis	9.5 (8.8;10.3)	48.3	40.0	2.5 [1.0–6.5]	7.7 [2.9–14.0]
Chronic back pain	6.7 (6.4;7.1)	89.3	96.7	2.0 [1.0–5.0]	4.5 [1.3–10.3]
Gout	1.6 (1.5;1.7)	--^e^	30.9	1.5 [1.0–4.0]	4.2 [1.1–10.4]
Osteoporosis	1.2 (1.1;1.4)	--^e^	38.5	3.5 [1.0–9.0]	5.7 [1.3–13.1]
Spondyloarthropathies	1.1 (0.9;1.3)	8.4	46.5	3.0 [1.0–7.5]	9.2 [3.9–14.8]
Rheumatoid arthritis	0.9 (0.8;1.0)	57.7	35.0	2.5 [1.0–7.5]	7.2 [2.1–14.1]

^a^ Considering the whole period since the first register available in the MedicineInsight dataset (2000–2016)

^b^ Consultations between Oct/2013 and June/2016

^c^ Number of registers on different dates when the patient received some diagnosis and/or some encounter occurred between the patient and the health service, including face-to-face visits and telephone contacts

^d^ Positives for arthritis, chronic back pain, gout, osteoporosis, spondyloarthropathies, and/or rheumatoid arthritis

^e^ No data available on diagnosis for these conditions before 2013

**Fig. 1:**
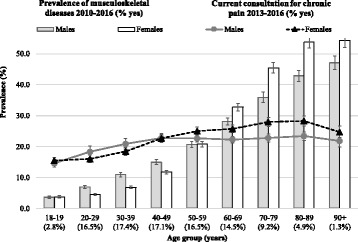
Overall prevalence of the investigated MSK and current consultation for chronic pain by age and sex. For “prevalence”, the whole period since the first register available in the MedicineInsight dataset (2000–2016) was considered, while “current consultation” considers the period between Oct/2013 and June/2016. Results for adults (18+ years) who attended one of the 329 Australian General Practices participating in the MedicineInsight program (*N* = 1,501,267). Vertical lines represent 95%CIs

Figure 1 shows that the “prevalence” of the investigated MSK and “current” consultations for chronic pain increased with age, but the last remained relatively stable for those aged 40 years or over, especially among males (Fig. 1). The “prevalence” of chronic MSK and “current” consultations for chronic pain were higher in males aged 20–39 years than in women of the same age, becoming both more frequent in women than in men after the age 60 years.

**Fig. 2:**
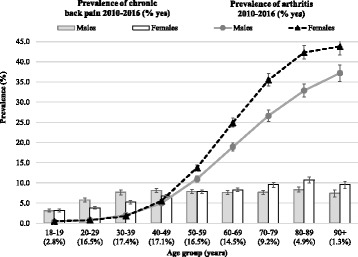
Prevalence of chronic back pain and arthritis by age and sex. “Prevalence” estimations considering the whole period since the first register available in the MedicineInsight dataset (2000–2016). Results for adults (18+ years) who attended one of the 329 Australian General Practices participating in the MedicineInsight program (*N* = 1,501,267). Vertical lines represent 95%CIs

The distribution of arthritis and chronic back pain according to age and sex (Fig. 2) reflects the differences previously described for the “prevalence” of MSK and “current” consultations for chronic pain. The “prevalence” of chronic back pain was 20–53% more common in males aged 20–49 years than their female peers, while no sex differences were found for arthritis in this age. On the other hand, the “prevalence” of arthritis increased after the age 50 years, especially among women, who were up to 29% more likely to have this condition than men. Moreover, although the “prevalence” of chronic back pain remained relatively stable at around 9% in older adults, it was 25–33% more frequent in females.

Table 2 presents the distribution of the overall prevalence of MSK and current consultations for chronic pain according to sociodemographic characteristics and smoking status. The overall “prevalence” of MSK ranged from 14.7% to 19.4% across states, except in NT where 10.2% had some of the investigated MSK. “Current” consultations for chronic pain oscillated around 20% in most states, but a higher frequency was observed in the ACT and the lowest in NT (26.1% and 8.8%, respectively). Consequently, “current” consultations for chronic pain among patients with some of the investigated MSK were more common in ACT (63.8%) and less frequent in NT (38.0%). Differences in the overall “prevalence” of MSK and “current” consultations for chronic pain were less evident for rurality or socioeconomic position (lower frequencies in remote Australia and in the highest SEIFA-IRSAD quintile), and a lower proportion of patients with some of the investigated MSK consulted for chronic pain in remote Australia. After adjustment for age and sex, the overall “prevalence” of MSK, “current” consultations for chronic pain, and proportion of patients with some of the investigated MSK consulting for chronic pain were higher in DVAs holders. Both, the overall “prevalence” of MSK and “current” consultations for chronic pain were more frequent in Aboriginal and Torres Strait Islander people. However, the proportion of consultations for chronic pain among those affected by some of the investigated MSK was similar in Aboriginals and non-Aboriginals. The overall “prevalence” of MSK and “current” consultations for chronic pain were similar in ex-smokers and current smokers, while among non-smokers these frequencies were lower.

**Table 2:** Prevalence of the investigated musculoskeletal diseases and consultations for chronic pain according to sociodemographic characteristics and smoking status. Adults aged 18+ years (*N* = 1,501,267)

	% of all data	Prevalence of chronic musculoskeletal disease (2010–2016)^a^		Current consultation for chronic pain (2013–2016)^b^		% of patients with a MSK that consulted for chronic pain (2013–2016)^b^ %
		%^c^	PR (95%CI)^c^	%^c^	PR (95%CI)^c^	
State			*P* < 0.001		*P* = 0.006	
New South Wales (NSW)	28.2	17.6	Ref	22.7	Ref	57.5
Victoria (VIC)	25.5	16.5	0.94 (0.85–1.04)	22.2	0.98 (0.84–1.14)	58.1
Queensland (QLD)	18.7	16.9	0.96 (0.88;1.06)	19.8	0.87 (0.77–0.99)	53.4
Western Australia (WA)	13.3	14.7	0.83 (0.72–0.96)	19.1	0.84 (0.72–0.99)	55.9
Tasmania (TAS)	8.0	19.4	1.10 (1.01–1.21)	21.8	0.96 (0.83–1.12)	52.8
South Australia (SA)	3.4	17.3	0.98 (0.87;1.11)	22.3	0.98 (0.82–1.17)	54.9
Australia Capital Territory (ACT)	1.6	18.8	1.07 (0.73;1.58)	26.1	1.15 (0.77–1.71)	63.8
North Territory (NT)	1.3	10.2	0.58 (0.39;0.88)	8.8	0.39 (0.22–0.67)	38.0
Rurality			*P* = 0.05		*P* = 0.07	
Major cities	63.0	16.4	Ref	21.4	Ref	57.2
Inner regional	24.2	18.1	1.11 (1.02–1.20)	22.4	1.05 (0.93–1.17)	55.5
Outer regional	11.2	16.9	1.03 (0.95–1.12)	19.4	0.91 (0.80–1.02)	52.4
Remote Australia	1.6	14.6	0.89 (0.71–1.12)	16.6	0.78 (0.61–1.00)	49.0
Socioeconomic position (SEIFA-IRSAD quintiles)			*P* < 0.001		*P* < 0.001	
1 (Lowest)	17.3	18.9	Ref	23.8	Ref	57.1
2	15.7	19.1	1.01 (0.93–1.08)	23.5	0.99 (0.90–1.08)	57.2
3	20.5	16.5	0.87 (0.80–0.94)	20.2	0.85 (0.77–0.93)	54.2
4	20.1	16.5	0.87 (0.81–0.94)	21.2	0.89 (0.81–0.98)	56.3
5 (Highest)	26.4	14.6	0.77 (0.71–0.84)	19.6	0.83 (0.74–0.93)	56.0
Pension			*P* < 0.001		*P* < 0.001	
None	22.9	13.3	Ref	17.8	Ref	51.5
Pensioner or health care card	32.6	21.7	1.64 (1.52–1.76)	26.0	1.46 (1.32–1.63)	59.0
Department of Veterans Affair	1.3	24.1	1.82 (1.68–1.98)	29.6	1.66 (1.47–1.88)	60.8
Ignored	43.2	14.3	1.08 (0.98–1.19)	20.3	1.14 (1.00–1.31)	55.1
Aboriginal or Torres Strait Islander			*P* < 0.001		*P* < 0.001	
No	69.7	17.6	Ref	22.2	Ref	56.9
Yes	1.8	22.9	1.30 (1.22–1.39)	25.8	1.16 (1.08–1.24)	58.2
Ignored	28.6	14.7	0.83 (0.79–0.88)	19.0	0.85 (0.80–0.91)	53.8
Smoking status			*P* < 0.001		*P* < 0.001	
Non-smoker	50.3	16.8	Ref	21.3	Ref	54.8
Ex-smoker	20.1	19.8	1.17 (1.15–1.19)	24.4	1.14 (1.12–1.17)	57.2
Current smoker	14.5	20.5	1.22 (1.19–1.25)	24.8	1.17 (1.12–1.21)	59.4
Ignored	15.1	10.0	0.65 (0.61–0.70)	15.7	0.69 (0.64–0.75)	54.4

^a^ Considering the whole period since the first register available in the MedicineInsight dataset (2000–2016), and including those positives for arthritis, chronic back pain, gout, osteoporosis, spondyloarthropathies and/or rheumatoid arthritis

^b^ Consultation between 10/2013 and 06/2016

^c^ Prevalence, prevalence ratios (PR) and p-values estimated based on Poisson Regression models, adjusted for sex and age. P-values of heterogeneity indicating differences across categories of each independent variable

**Fig. 3:**
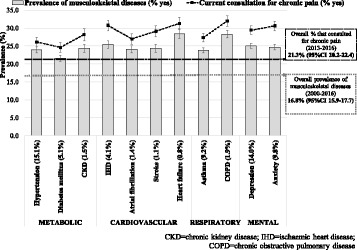
Overall prevalence of the investigated musculoskeletal diseases and current consultation for chronic pain according to the presence of chronic diseases. For “prevalence”, the whole period since the first register available in the MedicineInsight dataset (2000–2016) was considered, while “current consultation” considers the period between Oct/2013 and June/2016. Results for adults (18+ years) who attended one of the 329 Australian General Practices participating in the MedicineInsight program (*N* = 1,501,267). Values in parenthesis are the prevalence of each chronic disease. Vertical lines represent 95%CIs. Results are age and sex adjusted

Fig. 3 shows the overall “prevalence” of the investigated MSK and chronic pain among individuals affected by other chronic diseases. The investigated MSK were more frequent among those affected by heart failure or COPD, and less frequent among people with diabetes mellitus. On the other hand, “current” consultations for chronic pain among individuals with a chronic disease ranged from 25% to 29% for most diseases, with the highest values observed among those affected by ischaemic heart disease, heart failure, COPD, or anxiety.

## Discussion

Four principal findings can be highlighted in this study. Firstly, the investigated MSK and consultations for chronic pain are common, and often related, reasons for presentation in Australian general practice. Secondly, patients affected by some of the investigated MSK attend general practice more frequently than those without these conditions. Thirdly, although the overall “prevalence” of the investigated MSK increases with age, the rate of “current” consultation for chronic pain remains stable once patients reach 40–49 years of age, especially among males. Finally, there appears to be some geographical and socioeconomic differences in the overall “prevalence” of the investigated MSK and “current” consultations for chronic pain in Australia that need to be explained.

Previous estimates for the prevalence of MSK are similar to our “prevalence” obtained from MedicineInsight data [29], although we might expect a higher frequency in our study compared to community based surveys, because our participants are attending a doctor, which makes them less likely to be healthy than general population [17]. Using self-report data, the AIHW estimated that osteoarthritis affects 8.0% of all Australians adults, back pain 7.1%, osteoporosis 3.1%, and rheumatoid arthritis 1.9%. However, the use of self-reported information can undermine the ability to truly identify the scale of MSK within the population, especially for chronic pain, as the reliability of this information is also affected by question wording [15]. According to the BEACH program, chronic pain among Australian patients attending general practices changed from 18% in 2005 (*N* = 3211) to 19% in 2008–09 (*N* = 5793) [11, 26]. Therefore, the results obtained from MedicineInsight are similar to these previous estimates, with the advantage of providing results from a very large sample, which can be considered nationally representative of Australian adults (demographic distribution comparable to the census) [17, 30]. It is also based on medical diagnosis rather than self-report. Different countries have demonstrated that data from primary health care electronic records are accurate for the investigation of different diseases and their risk factors (sensitivity, specificity, and predictive positive values ranging from 70 to 95%) [19–23]. Programs like MedicineInsight are an excellent opportunity for governments to ascertain clinical outcomes on a large scale, at low cost, and with the potential to link to other data sources, thus facilitating monitoring and planning by heath policy makers [31]. Our findings also highlight the importance of these conditions in Australian health care because of their chronicity, rising health care costs, higher frequency among individuals affected by other chronic conditions, and adverse effects on patients’ quality of life [6, 7, 32, 33].

It has been shown previously that MSK are a common reason for consultation in general practice, increasing with age and being more frequent in females [10, 34]. Chronic pain has also been reported to be more frequent among the less educated, those with lower family income, the physically inactive, smokers, and among those with depression [29], which is consistent with our results. Additionally, some studies have also found a higher frequency of chronic pain among women and a progressive increase with age, with a reduction after the age of 65 years, which is also consistent with our findings [11, 29, 35]. The reduction in the “prevalence” of chronic pain in the elderly may be a consequence of fewer work place related physical adverse effects post retirement [29, 35]. However, this could be also secondary to GPs recording MSK more frequently than chronic pain as the reason for encounter once they confirm the diagnosis, or given the increase in comorbidity with age, the reporting of chronic pain may slip down the list of priorities compared to the other conditions older people have. However, some biological explanations are also possible. For instance, MSK developed later in life may be less painful due to a higher pain threshold associated with a loss of primary afferent fibres, reduction of pain receptors, or pain indifference resulting from a history of other chronic problems [36, 37].

More difficult to explain are the differences in consultation rates for chronic pain related to MSK between States, which has not been reported before in Australia [6, 11, 12, 32, 34], and may relate to differential access to general practice (i.e. poor access in non-urban locations) or recording of the reason for consultation, such as in the NT, where the percentage of excluded individuals due to missing data was 10.7%.

An advantage of using routinely collected morbidity data is the ability to examine the relationship between conditions. As expected, those with anxiety were more likely to consult for chronic pain [38]. Amongst co-morbidities, the overall prevalence of the investigated MSK and chronic pain were particularly high among those affected by heart failure and COPD. This relationship has been reported previously [39–42] and attributed to abnormal inflammatory responses in extra-articular tissues, co-morbid depression and anxiety, insomnia, related symptoms (i.e. cough making pain worse, oedema), musculoskeletal fatigue, and restricted use of non-steroidal anti-inflammatory drugs for pain management.

In the absence of a disease register for MSK in general practice, MedicineInsight provides an alternative to population surveys. However, there are some limitations that should be highlighted. Firstly, not everyone with a MSK needs to see a GP regularly for diagnosis or management. For instance, most people over the age of 65 will have osteoarthritis or some other MSK, but not everyone will require regular medical care for these conditions [6, 7]. Secondly, depending on how critical information is recorded (e.g. missing data, non-mandatory fields, free-text for coding, different system coding vocabularies) or extracted (algorithms for data extraction), the accuracy of the information may be compromised [20–23, 43]. Although 2.8% of the individuals were excluded due to missing data on diagnosis or reason for encounter, it is very unlikely that it could have biased the results, as they were similar to the investigated sample according to sex, age, rurality, socioeconomic position, aboriginality or smoking status. Even though diverse methods were used to improve data quality (i.e. general practice characteristics, fields used for data extraction, variability of terms and synonyms), the algorithms used by MedicineInsight for data extraction have not been validated [28]. Furthermore, considering the non-random sampling process used for the enrolment of general practices and restrictions defined by MedicineInsight to get them included in the datasets, smaller practices are more likely to be underrepresented. However, the investigated sample seems to be representative of the Australian population, as the distribution in terms of gender, age, socioeconomic position, state, and rurality closely resembles figures from the last census [17, 30]. In any case, considering all these issues, MedicineWise is continuously expanding the number of recruited general practices and working towards the standardisation of its own data coding and extraction procedures [17]. Thirdly, diseases that are longstanding may only be recorded at initial diagnosis and a snapshot of GP attendance may not pick up the sentinel event. Including a longer period of data collection for the same patients and using a list of synonyms for the investigated conditions could improve the sensitivity to identify MSK cases and overcome these limitations.

## Conclusions

The investigated MSK were an important and frequent reason for consultation in Australian general practice, especially among lower socioeconomic groups and the elderly. The distribution of these conditions differed according to other demographic characteristics and presence of comorbidities. Based on information collected from adults attending Australian general practices, MedicineInsight provided similar estimates to those obtained from population-based studies, with the advantage of being based on medical diagnosis and including a national sample.

## Additional files

Additional file 1: Figure S1. Venn diagram for the combined frequency of the investigated musculoskeletal diseases and current consultation for chronic pain. Venn diagram for the combined frequency of the investigated musculoskeletal diseases and current consultation for chronic pain. For “prevalence”, the whole period since the first register available in the MedicineInsight dataset (2000-2016) was considered, while “current consultation” considers the period between Oct/2013 and June/2016. Results for adults (18+ years) who attended one of the 329 Australian General Practices participating in the MedicineInsight program (N=1,501,267). (DOC 218 kb)

## Abbreviations

ACT: Australia Capital Territory
AIHW: Australian Institute of Health and Welfare
BEACH: Bettering the Evaluation and Care of Health Program
COPD: Chronic obstructive pulmonary disease
DVA: Department of Veterans Affair
GPs: General practitioners
MSK: Musculoskeletal conditions
NCDs: Chronic non-communicable diseases
NHS: National Health Survey
NPS: National Prescribing Service
NT: North Territory
PMH: Past medical history
PR: Prevalence ratio
SEIFA-IRSAD: Australian socio-economic indexes for areas index of relative socio-economic advantage and disadvantage
TAS: Tasmania
WA: Western Australia
