# Supplementary material for: Epidemiology of arthritis, chronic back pain, gout, osteoporosis, spondyloarthropathies and rheumatoid arthritis among 1.5 million patients in Australian general practice: NPS MedicineWise MedicineInsight dataset
Source: BMC Musculoskelet Disord. 2018 Jan 18;19:20. doi: 10.1186/s12891-018-1941-x (PMC5774097; doi:10.1186/s12891-018-1941-x)
Supplement: Supplementary file 1 — Venn diagram for the combined frequency of the investigated musculoskeletal diseases and current consultation for chronic pain. Venn diagram for the combined frequency of the investigated musculoskeletal diseases and current consultation for chronic pain. For “prevalence”, the whole period since the first register available in the MedicineInsight dataset (2000-2016) was considered, while “current consultation” considers the period between Oct/2013 and June/2016. Results for adults (18+ years) who attended one of the 329 Australian General Practices participating in the MedicineInsight program (N=1,501,267). (DOC 218 kb) [file 12891_2018_1941_MOESM1_ESM.doc]

**Available sample in the MedicineInsight database**

**N = 1,501,267 adults**

**Current consultation for**

**chronic pain 2013-2016**

**(21.3%)**

**Positive for some of**

**the investigated MSK**

**2010-2016**

**(16.8%)**

**Negative for chronic pain or MSK**

**[71.3 %]**

**Both**

**[9.4%]**

[% of total]

**Just chronic**

**pain**

**[11.9%]**

**Just a**

**MSK**

**[7.1%]**
